# Supplementary material for: Using Bayesian statistics in confirmatory clinical trials in the regulatory setting: a tutorial review
Source: BMC Med Res Methodol. 2024 May 7;24:110. doi: 10.1186/s12874-024-02235-0 (PMC11077897; doi:10.1186/s12874-024-02235-0)
Supplement: Supplementary file 1 — Supplementary Material 1. [file 12874_2024_2235_MOESM1_ESM.pdf]

# Supplemental Material of "Using Bayesian Statistics in Confirmatory Clinical Trials in the Regulatory Setting: A Tutorial Review"

## 1 An algorithm to approximate the power function

An algorithm employing nested simulation technique to approximate the power function

$$\begin{aligned}\psi(\theta) &= \mathbb{P}_\theta[T(\mathbf{y}_N) > \lambda] = \mathbb{P}[T(\mathbf{y}_N) > \lambda | \mathbf{y}_N \sim f(y|\theta)] \\ &= \int \mathbf{1}\{T(\mathbf{y}_N) > \lambda\} \prod_{i=1}^{n} f(y_i|\theta) d\mathbf{y}_N. \end{aligned} \tag{1}$$

is given as follows:

## 2 Proof of Theorem 1

Let $\theta_t$ denote the true data generating parameter. Due to the asymptotic normality of the maximum likelihood estimator, if the sample size $N$ is sufficiently large, then the maximum likelihood estimator $T_1(\mathbf{y}_N)$ is normal with mean $\theta_t$ and variance $1/I(\theta_t)$, where $I(\theta) = \mathbb{E}[(\partial^2/\partial\theta^2) \log \ f(\mathbf{y}_N|\theta)]$ denotes the Fisher information. By setting the performance goal $\theta_0$ as the truth $\theta_t$, we can express the p-value of the frequentist test procedure as follow:

$$\begin{aligned} p(\mathbf{y}_N) &= \mathbb{P}\left[T_1(\mathbf{y}_N^{rep}) > T_1(\mathbf{y}_N) | \mathbf{y}_N^{rep} \sim f(y|\theta_0)\right] \\ &= \mathbb{P}\left[\sqrt{I(\theta_0)} \cdot (T_1(\mathbf{y}_N^{rep}) - \theta_0) > \sqrt{I(\theta_0)} \cdot (T_1(\mathbf{y}_N) - \theta_0) | \mathbf{y}_N^{rep} \sim f(y|\theta_0)\right] \\ &\approx \mathbb{P}\left[Z > \sqrt{I(\theta_0)} \cdot (T_1(\mathbf{y}_N) - \theta_0) | \mathbf{y}_N^{rep} \sim f(y|\theta_0)\right] \quad \text{for large } N \\ &= \mathbb{P}\left[Z < \sqrt{I(\theta_0)} \cdot (\theta_0 - T_1(\mathbf{y}_N)) | \mathbf{y}_N^{rep} \sim f(y|\theta_0)\right] \\ &= \Phi\left(\sqrt{I(\theta_0)} \cdot (\theta_0 - T_1(\mathbf{y}_N))\right), \end{aligned} \tag{2}$$

where $Z$ and $\Phi(x)$ represent the random variable and cumulative distribution function of standard normal distribution, respectively. $\mathbf{y}_N^{rep}$ represents a hypothetical representation of data $\mathbf{y}$ under the null distribution.

**Algorithm 1:** A nested simulation to approximate the power function $\psi(\theta)$

**Goal:** Approximating the power function $\psi(\theta)$ (1) evaluated at $\theta \in \Theta$.
**Input:** A prior $\pi(\theta)$, data generating distribution $f(y|\theta)$,
threshold value $\lambda \in [0,1]$, Bayesian test statistics $T(\mathbf{y}_N)$,
true data generating parameter $\theta$, number of repetitions of trials $R$,
and number of posterior samples $S$.
**Output:** An approximated value $\widetilde{\psi}(\theta)$.
**For** ( $r = 1, \cdots, R$ ) **{**

- Generate the synthetic responses of $N$ patients assuming that $\theta$ is true

$$\mathbf{y}_N^{(r)} = (y_1^{(r)}, \cdots, y_N^{(r)}) \sim f(y|\theta)$$

**For** ( $s = 1, \cdots, S$ ) **{**

- Sample from posterior distribution given $\mathbf{y}_N^{(r)}$

$$\theta^{(s)} \sim \pi(\theta|\mathbf{y}_N^{(r)})$$

**}**

- Approximate Bayesian test statistics $T(\mathbf{y}_N^{(r)})$ by $\{\theta^{(s)}\}_{s=1}^S$

$$\widehat{T}(\mathbf{y}_N^{(r)}) = g(\theta^{(1)}, \cdots, \theta^{(S)}),$$

where the function $g(\cdot)$ is chosen appropriately according to the form of $T(\mathbf{y}_N)$

**}**

- Approximate power function $\psi(\theta)$ by $\{\widehat{T}(\mathbf{y}_N)^{(r)}\}_{r=1}^R$

$$\widetilde{\psi}(\theta) = \frac{1}{R}\sum_{r=1}^{R} 1\left(\widehat{T}(\mathbf{y}_N^{(r)}) > \lambda\right)$$

On the other hand, the Bernstein-Von Mises theorem [1, 2] states that, if the sample size $N$ is sufficiently large, the posterior distribution $\pi(\theta|\mathbf{y}_N)$ is approximately normally distributed with the mean same as $T_1(\mathbf{y}_N)$ (the maximum likelihood estimator) and the variance same as the reciprocal of the Fisher information evaluated at the truth, i.e., $1/I(\theta_t)$, independently of the form of prior $\pi(\theta)$. Thus, the following equation holds

$$\begin{aligned} T_2(\mathbf{y}_N) &= \mathbb{P}\left[\theta > \theta_0 | \mathbf{y}_N\right] \\ &= \mathbb{P}\left[\sqrt{I(\theta_t)} \cdot (\theta - T_1(\mathbf{y}_N)) > \sqrt{I(\theta_t)} \cdot (\theta_0 - T_1(\mathbf{y}_N)) | \mathbf{y}_N\right] \\ &\approx \mathbb{P}\left[Z > \sqrt{I(\theta_t)} \cdot (\theta_0 - T_1(\mathbf{y}_N)) | \mathbf{y}_N\right] \quad \text{for large } N \\ &= 1 - \Phi\left(\sqrt{I(\theta_t)} \cdot (\theta_0 - T_1(\mathbf{y}_N))\right). \end{aligned}$$

Therefore, if the true data generating parameter $\theta_t$ is the performance goal $\theta_0$, it holds

$$T_2(\mathbf{y}_N) \approx 1 - \Phi\left(\sqrt{I(\theta_0)} \cdot (\theta_0 - T_1(\mathbf{y}_N))\right) \quad \text{for large } N. \tag{3}$$

By the asymptotic equations (2) and (3), it holds $T_2(\mathbf{y}_N) \approx 1 - p(\mathbf{y}_N)$ if $N$ is sufficiently large.

# References


[1] Johnstone, I.M.: High dimensional bernstein-von mises: simple examples. Institute of Mathematical Statistics collections **6**, 87 (2010)

[2] Walker, A.M.: On the asymptotic behaviour of posterior distributions. Journal of the Royal Statistical Society Series B: Statistical Methodology **31**(1), 80–88 (1969)
